# Emerging Trends in the Multimodal Nature of Cognition: Touch and Handedness

**DOI:** 10.3389/fpsyg.2017.00844

**Published:** 2017-05-26

**Authors:** Miriam Ittyerah

**Affiliations:** Department of Psychology, University of DelhiNew Delhi, India

**Keywords:** lateralization, hand ability, haptics, multimodal, tactile cognition

## Abstract

Advances in tactile cognition and haptics have increased our understanding of the multimodal nature of touch. Haptic data is mostly confined to human performance arising from the flexibility and dexterity of the fingers used to discriminate shapes and objects. Studies with infants indicate that recognition of objects either seen or held in the hand is possible during early periods of infancy. Evidence indicates performance differences between the hands decrease over periods of development, reflecting maturation of the cortical brain system supporting motor skills. Thus ability is not confined to the preferred hand. Tactile process and haptic cognition reflect hand ability. Studies examining manual performance must consider the relevance of haptics in research. Knowing about the evolution of the hands controlled by the cerebral hemispheres is of interest because it is a major contribution to the repertoire of human hand actions. The emergence of RDBM (role differentiated bimanual manipulation) is an important shift in the development of infant manual skills. Between 4 and 7 months of age, infants begin to manipulate objects using RDBM where one hand stabilized an object while the other hand manipulated the object. Understanding the affordance of a tool is an important cognitive milestone in early sensorimotor period that develops during the second year in full-term infants. This ability has also been demonstrated in preterm infants indicating the emergence of handedness during prenatal periods. Thus a multimodal approach that incorporates studies of tactile processes and hand actions may reveal their interactions with task demands and haptic ability.

## Introduction

The subject of cognition in Psychology has evolved from Gestalt views of brain isomorphism to attributing information processing capacities and mental rotation abilities in the brain (Miller, 1956; Shepard and Metzler, 1971). Studies of mental rotation have influenced studies in mental imagery while generating the imagery debate (Kosslyn, 1980). It is now known that imagery is not only visual or auditory. Tactile imagery in movement form can be represented in totally blind conditions (Millar and Ittyerah, 1991). It is of interest to discuss the growing importance of haptic touch and cognition and its inherent connection to hand preference and ability. The reason we know little concerning haptic cognition in comparison to visual or auditory cognition is that in contrast to other senses, animal species are not comparable to humans with regard to haptics. This seems to be an underestimated difference in animal-human comparisons. A further interesting phenomenon is that in contrast to other senses, motor skills play a major role in haptics for both executing an action and also using the hand as a sensory organ to scan a surface.

## Haptic Cognition and Hand Ability

The study of haptics or active movement in touch (Millar, 1994, 2008) has attained an interdisciplinary status. The haptic system involves two functional characteristics simultaneously: it processes the spatial properties and carries out this task in a sequential manner (Hatwell et al., 2003). These functional properties make its study complex. The tactile perception of an object is more accurate with systematic than unsystematic exploration. Accurate haptic coding of information is dependent upon reference frames. In blind conditions body centered frames of reference have been used reliably to perform tabletop tasks (Millar, 2008). Table top tasks such as aiming and movement require the subject to move the hand held with a pencil from a start to a goal point drawn as a path on paper, with different land marks, such as home, post office. Subjects are expected to reproduce a moved extent correctly, as the path from home to bus stop. Such tasks performed in blind conditions indicate that haptic distance judgments are not solely kinesthetic inputs and movement distances can be coded spatially if they are related to reference information as the body midline (Millar and Al-Attar, 2003a). These effects can be obtained independently with each hand even if the hands have an inherent specialization such as better movement processing with the right hand or better spatial detection with the left hand (De Renzi, 1978, 1982). Millar and Al-Attar (2003b) demonstrated that the left and right hands did not differ from each other in the control or the reference instruction conditions. The added reference information in the experimental condition significantly reduced errors, regardless of whether the left hand scanned the distance and the right hand was used for the frame, or whether the right hand scanned the distance and the left hand was used for the frame. Thus general laterality does not affect ability (Ittyerah, 2013), as is evident in the tactile discrimination of bricks by congenitally blind children independently with their left and right hands, (Ittyerah, 1993) and sorting and stacking objects (Ittyerah, 2013). Lateralization increases with age in development (McManus et al., 1988; Ittyerah, 1993) in both congenitally blind and sighted children and hand preference is not an indication of hand ability. With each hand controlled by the contralateral hemisphere, indicating specialization of actions within each hemisphere, findings show that left and right side performance differences decreased over development (Roeder et al., 2008) in children between 7 and 14 years on timed unimanual and unipedal tasks, reflecting the myelination and maturation of the cortical systems supporting motor skills.

Furthermore evidence indicates preattentive sensory processes during tactile searches as in vision, when stimulus characteristics such as sharp edges or angles pop out during contact (Plaisier et al., 2008). Plaisier et al. (2008) found that while haptic discriminations were performed between different sizes and shapes of objects such as cubes or cylinders in the hand, certain stimulus characteristics as sharp edges or contours seem to be distinct or pop out to enable effective stimulus feature discrimination. In addition, numerosity estimation or subitizing in touch revealed that the haptic system can effectively subitize three items with the left and the right hands equally well, and the point at which subitizing ends and counting begins is between three and four items (Plaisier et al., 2010). Thus automatic outcomes such as pop out of features and subitizing considered to be predominantly visual have also been observed in the haptic sense. Studies with infants indicate both visual and tactile recognition of objects during early periods of infancy (Hatwell, 2003). Using an intersensory paired preference procedure, Streri and Gentaz (2003) observed that newborns can visually recognize the shape of an object that they have previously manipulated with their right hand, out of sight. The hands are also motor organs used to reach, hold, transform or transport objects. Thus the hands are a complex system that involves two functions: a perceptual function (knowing) and an instrumental function (doing) (Streri, 2005).

## Lateralization and Performance

Knowing about the evolution of hand use is important because it is a major contribution to existing lateralization and the repertoire of human hand actions. It is now established that brain lateralization is no longer confined to the human brain but exists among animals or the vertebrate species (Rogers and Andrew, 2002) and among invertebrates (Frasnelli, 2013). Usually forelimb preferences have been studied among animals (Versace and Vallortigara, 2015) showing that selective pressures for different functions such as tool-use, communication, bipedal posture, task complexity, have likely influenced the evolution of forelimb preferences. Recent evidence shows that nearly 70% of non-human vertebrates show limb preference (Ströckens et al., 2013). It is of interest therefore to incorporate findings of animal species to know about the evolution of vertebrate lateralization and how this is comparable to lateralization in humans. Of particular interest are the homologies in cerebral hemispheres that may exist between different animal vertebrate species and humans.

The division of labor by the two hemispheres predates humankind by half a billion years (MacNeilage et al., 2009). These authors for example argue that cerebral hemispheres in less evolved vertebrates like the chick have been long specialized and reveal local and global processing behaviors. Local processing refers to the detection of features of a stimulus such as better grain pecking seen with the right eye of the chick controlled by the left hemisphere, whereas global processing refers to better detection and response to unexpected stimuli with the left eye controlled by the right hemisphere, attending to global aspects in the environment. Similar functional differences between the hemispheres among human subjects were demonstrated (Navon, 1977) when task instructions influenced the perception of global or local stimulus characteristics that are predominantly controlled by the right and left hemispheres. MacNeilage et al. (1987), observed that the hands of prosimians showed some specialization such as grasping for support with the right hand (left hemisphere) and striking prey with the left hand (right hemisphere), revealing task demands for the obligate use of a particular hemisphere. It has been suggested that the left hemisphere develops before the right hemisphere (MacNeilage et al., 2009; Fagard, 2013) and thus a lateralized brain pre-empts hand use. These findings have been interpreted as signs of laterality in chimpanzees when they use each hand separately to complete flint puzzles equally well and a complementary role differentiation between the hands for bimanual actions of nut cracking, indicating tasks vary in their gradient of laterality (Uomini, 2009).

Recent studies show that there may be subtle functional differences between the hands (Schabowsky et al., 2007; Sainburg, 2014). These authors provide a structure for understanding the motor control process that give rise to handedness. In their dynamic dominance model, the left hemisphere in right handers was proficient for processes that predict the effects of body and environmental dynamics, whereas the right hemisphere was proficient at impedance control and had better final position accuracy. Chronic stroke patients with left or right hemisphere damage, using their ipsilesional/unaffected arm in ipsilesional space, performed reaching movements that conform to the dynamic dominance model (Schaefer et al., 2009), indicating each hemisphere contributes different control processes to each arm. Left hemisphere damage group produced deficits in controlling the ipsilesional arm trajectory, whereas the right hemisphere damage group showed deficits in ipsilesional final position accuracy. These findings display complementary actions and ability of both hands. We (Ittyerah et al., 2007) have demonstrated that the difference between the hands is in the orientation that each hand adopts during a task and not in ability. Congenitally totally blind children and sighted blindfolded children were able to point at targets in space on a sagittal screen in the absence of vision. The right hand adopted a context oriented approach whereas the left hand adopted an egocentric approach. The egocentric orientation increased pointing error in the left hand to indicate orientation rather than ability differences between the hands. Stone and Gonzalez (2015b) observed that the right hand controlled by the left hemisphere was dominant for visually guided grasping and the left hand/right hemisphere was dominant for haptically guided object recognition. In a block building task without vision, sighted blind folded children showed a marked decrease in the right hand use when vision was unavailable suggesting haptics can be used to guide reaching and grasping as early as 5 years of age (Stone and Gonzalez, 2015a).

By 4 months of age there is a division of labor between the hands, revealing better right hand performance for fine motor movements and left hand performance for spatial arrangement and haptic processing (Morange-Majoux et al., 1997; Morange-Majoux, 2011). These findings are in conformity with those of Rogers and Andrew (2002) and Sainburg (2014) who indicated that the right hand controlled by the left hemisphere controls environmental dynamics or local effects and the right hemisphere controlled by the left hand controls for impedance or global effects such as the better detection and response to unexpected stimuli. However, Morange-Majoux’s studies were designed to test for reaching and processing objects during infancy. Four to six months infants spent more time with the left hand in the haptic exploration of cylinders (Morange-Majoux, 2011). Some authors concluded that increased time spent touching the object with the left hand is due to deeper haptic information processing ability of the left hand (Lhote and Streri, 1998). Furthermore between 4 and 7 months of age, infants begin to manipulate objects using role-differentiated bimanual manipulation (RDBM) where one hand stabilized an object while the other hand manipulated the object (Rochat, 1989; Kimmerle et al., 2010).

The emergence of RDBM is an important shift in the development of infant manual skills. In bimanual tasks differing in the precision of the movement required to remove pieces of paper from a stalk, the right hand was used more often than the left not only to grasp the object but also to remove pieces. Though infants do not anticipate the need of their preferred hand to remove pieces, they showed handedness in these coordinated bimanual tasks to a greater degree on those requiring precision grips than whole hand grip, indicating handedness develops early and is related to the precision required from the active hand (Potier et al., 2013). Understanding the affordance of a tool is an important cognitive milestone in early sensorimotor period. Using a tool to bring within reach an out-of-reach object has been shown to develop during the second year in full-term infants. Petkovic et al. (2016) presented preterm infants with an attractive toy out of reach and with a rake-like tool within reach in five conditions of spatial relationships between the toy and the tool. Like full-terms, preterm infants used the tool with success in conditions of spatial contiguity around 15–17 months. In conditions of a spatial gap between tool and toy, preterm infants as a group showed no delay for tool use indicating handedness emerges during the prenatal stage. Thus early in infancy, the predominance of touch/haptics over vision and a hand preference equips the infant for successful transactions in the environment.

In summary a multimodal approach that incorporates studies of tactile processes and hand actions may clarify interactive processes in the sense modality of touch. Task demands require interactive processes that are a combination of thought, movement and hand actions to produce the required response. Although tactile sensations are perceived during contact, lateralization and ability of the hand is reflected in the nature of the grip and force applied to the object by its fingers. The problem of producing devices that function like the human hand is mixed with challenges and insights. Since haptics has recently become important in robotics the results of such human-centered studies could be revealing.

## Author Contributions

The author confirms being the sole contributor of this work and approved it for publication.

## Conflict of Interest Statement

The author declares that the research was conducted in the absence of any commercial or financial relationships that could be construed as a potential conflict of interest.
